# Medical problems: review from the major nuclear incidents

**DOI:** 10.1186/cc12426

**Published:** 2013-03-19

**Authors:** Y Haraguchi, Y Tomoyasu, H Nishi, M Hoshino, M Sakai, E Hoshino

**Affiliations:** 1 Disaster Medicine Compendium Team, Japan, Tokyo, Japan; 2 Shirahigebashi Hospital, Tokyo, Japan; 3 Tokyo Disaster Medical Center, Tokyo, Japan; 4 Shisei Hospital, Saitama, Japan; 5 Tokyo Women's Medical College, Tokyo, Japan; 6 Chiba Nursing Association, Chiba, Japan

## Introduction

Intensivists are expected to have many roles during and after a major disaster/catastrophe; that is, triage, intensive care, education for people, and so forth. The roles of intensivists against special disaster or nuclear disaster are studied based on actual experiences.

## Methods

Several disasters are studied. The Fukushima Daiichi Nuclear plant explosion after the Higashinihon earthquake 2011 was medically reviewed based on the total 30-day stay on-site in addition to several days around the site. The Chernobyl incident 1986 was inspected 15 years after the incident. Other nuclear disasters are included.

## Results

Many serious problems were revealed in the medical teams, which are as follows: inappropriate basic preparedness against large special disasters, including nuclear disaster; lack of appropriate education and training for medical teams against nuclear disaster - that is, most members of Japan DMAT or the disaster medical assistance team are still laypersons; incorrect standard/rules of Japan DMAT, which were excessively focused upon cure of the usual type of injury and planned short period or nearly 48 hours, which should be abandoned; and insufficient consideration to the weak/vulnerable people or CWAP, children, (pregnant) women, aged people, and the poor people/sicker patients. Many of them died because of an insufficient emergency transportation system from their contaminated houses or hospital.

## Conclusion

In order to cope with the special disasters, such as NBC or nuclear, biological and chemical disaster, it is insufficient to take makeshift measures or use cheap tricks. Working out the systematization of disaster medicine, based upon the academic viewpoints and philosophy/reliability, is essential to protect the people and the nation.

